# Сhronotype and health related quality of life among undergraduate university students

**DOI:** 10.1192/j.eurpsy.2024.1395

**Published:** 2024-08-27

**Authors:** E. L. Nikolaev, I. Poverinov, M. Alhasan

**Affiliations:** ^1^Department of Social and Clinical Psychology; ^2^Philosophy, Sociology and Pedagogy Department; ^3^Medical Faculty, Ulianov Chuvash State University, Cheboksary, Russian Federation

## Abstract

**Introduction:**

Chronotype represents genetically determined behavioral characteristics of a person’s twenty-four-hour activity. Research shows that a person’s chronotype is interrelated with their mental health. Are there similar connections with general health and health related quality of life?

**Objectives:**

To establish how various chronotypes are represented in university students and if there are any interrelations between chronotypes and health‐related quality of life

**Methods:**

We used SF-12 Health Survey и Morningness–Eveningness Questionnaire (MEQ) by Horne and Ostberg to survey 305 university students of both genders.

**Results:**

The results showed that the majority of the students (71.2%) have an intermediate chronotype. The second goes a moderate morning chronotype (17.7%), the third – a moderate evening chronotype (9.8%). Definite morning and definite evening chronotypes were revealed in less than 1% of the students. SF-12 Health Survey scale indicators that assess quality of life corresponded to standard scores for the given group of the respondents. We have revealed valid relations in correlational interconnections of the achieved parameters. Thus, the morning chronotype is most consistently associated (p<0.01) in undergraduate university students with higher indicators of health related quality of life including General Health (r=.23), Vitality (r=.21), Role Physical (r=.18), Role Emotional (r=.17), Physical Functioning (r=.16), Social Functioning (r=.13). Mental Health and Bodily Pain in university students are not connected with the chronotype (p>05).

**Conclusions:**

Therefore, this research establishes that the majority of the students are related to the intermediate chronotype, and the morning chronotype corresponds to higher levels of most indicators of health related quality of life excluding the level of mental health.

**Disclosure of Interest:**

None Declared

